# Characterising food environment exposure at home, at work, and along commuting journeys using data on adults in the UK

**DOI:** 10.1186/1479-5868-10-85

**Published:** 2013-06-27

**Authors:** Thomas Burgoine, Pablo Monsivais

**Affiliations:** 1 UKCRC Centre for Diet and Activity Research (CEDAR), Box 296, Institute of Public Health, Forvie Site, Robinson Way, University of Cambridge, Cambridge CB2 0SR, UK; 2 Department of Public Health and Primary, University of Cambridge, Cambridge, UK

**Keywords:** Food environment, Neighbourhood, Home, Work, Commuting, Exposure assessment, Geographic information systems, Density, Proximity

## Abstract

**Background:**

Socio-ecological models of behaviour suggest that dietary behaviours are potentially shaped by exposure to the food environment (‘foodscape’). Research on associations between the foodscape and diet and health has largely focussed on foodscapes around the home, despite recognition that non-home environments are likely to be important in a more complete assessment of foodscape exposure. This paper characterises and describes foodscape exposure of different types, at home, at work, and along commuting routes for a sample of working adults in Cambridgeshire, UK.

**Methods:**

Home and work locations, and transport habits for 2,696 adults aged 29–60 were drawn from the Fenland Study, UK. Food outlet locations were obtained from local councils and classified by type - we focus on convenience stores, restaurants, supermarkets and takeaway food outlets. Density of and proximity to food outlets was characterised at home and work. Commuting routes were modelled based on the shortest street network distance between home and work, with exposure (counts of food outlets) that accounted for travel mode and frequency. We describe these three domains of food environment exposure using descriptive and inferential statistics.

**Results:**

For all types of food outlet, we found very different foodscapes around homes and workplaces (with overall outlet exposure at work 125% higher), as well as a potentially substantial exposure contribution from commuting routes. On average, work and commuting environments each contributed to foodscape exposure at least equally to residential neighbourhoods, which only accounted for roughly 30% of total exposure. Furthermore, for participants with highest overall exposure to takeaway food outlets, workplaces accounted for most of the exposure. Levels of relative exposure between home, work and commuting environments were poorly correlated.

**Conclusions:**

Relying solely on residential neighbourhood characterisation greatly underestimated total foodscape exposure in this sample, with levels of home exposure unrelated to levels of away from home exposure. Such mis-estimation is likely to be expressed in analyses as attenuated parameter estimates, suggesting a minimal ‘environmental’ contribution to outcomes of interest. Future work should aim to assess exposure more completely through characterising environments beyond the residential neighbourhood, where behaviours related to food consumption are likely to occur.

## Background

Socio-ecological models of behaviour suggest that dietary behaviours are potentially shaped by exposure to the food environment (‘foodscape’) [1,2]. The foodscape, composed of a mix of retail food outlets such as supermarkets and restaurants, can promote either healthy or unhealthy dietary choices, mediated through and modified by individual and household sociodemographic, economic and psychological factors [3,4]. However, despite recent theoretical, methodological and analytical advances [5-10], the research on neighbourhood associations with diet and health has largely focused on foodscapes around the home. The significance of non-home environments to diet and health has been recognised [11-13], with the assessment of these environments likely to be especially important for understanding dietary behaviours in working age adults [14,15].

Recent studies have suggested that non-home foodscape exposures could contribute substantially to ‘exposure truth’ [16]. One study found that 49% of participants in Seattle had greater supermarket exposure when outside of the home neighbourhood [17], whilst another observed that fast food outlet exposure around workplaces was more than two-fold higher than around residences [18]; similar results have been found elsewhere [19,20]. Considering supermarkets as ‘enablers’ of a healthy diet [21,22], and the relative unhealthiness of fast foods consumed away from the home [23], these non-home exposures may be particularly important for food intake and health. In one study, fast food density around work but not around the home was associated with BMI [18], attesting to the importance of workplace exposure. Had foodscape exposure been based on residential exposures only, the exposure classification of study participants would have been biased [9], limiting the ability to accurately detect associations between environment and BMI. Importantly, relative levels of foodscape exposure in home and non-home environments need to be more fully explored. Exposure to relatively different levels of exposure in distinct environmental domains may be particularly problematic in ensuing analyses. For example, associations between relatively low home food outlet exposures and diet may be substantially confounded by relatively high exposures in non-home domains.

Travel or commuting routes are another foodscape exposure that has not been well characterised, perhaps due to the challenges of accurately capturing individual mobility patterns [9]. One study focussed on locations (other than the home) visited throughout the day, and found elevated exposure to restaurants [11] across this home/non-home divide, but did not study potential foodscape exposure whilst travelling *per se*. Another study found elevated fast food outlet exposure whilst travelling throughout the day, but did not allow potential exposure to vary according to travel preferences [24]. Despite debate over the accuracy of doing so [25], recent studies of children’s environments have largely used imputed (shortest) routes between homes and schools to examine foodscape exposure [19,26,27]. Again, these studies have not accounted for transport mode preferences of participants or the frequency of use of different transport modes, which are likely to impact directly upon both route taken and degree of potential exposure along a given route, however they represent an important attempt to include a new dimension to a more complete estimate of environmental exposure.

This study examines foodscape exposure in common and salient foodscape domains – homes, workplaces and home-work commuting routes (accounting for travel preferences) – using a sample of working adults in Cambridgeshire, UK. Our aims were: 1) to detail the data sources and methods used to derive estimates of foodscape exposure, which extend beyond the residential address, and include travel mode and frequency; 2) to describe and test for significant differences in the distributions of food outlets by type (using a number of exposure metrics) in each domain, and to examine domain specific contributions to total foodscape exposure, and; 3) to assess whether relative levels of food environment exposure across the three domains were similar on a per-person basis.

## Methods

### Sample characteristics

The Fenland Study began in 2005 and is a population-based investigation into lifestyle and health, conducted by the Medical Research Council Epidemiology Unit in Cambridgeshire, UK. At the time of this study, the full Fenland study sample included 6,379 adults aged 29 to 60, recruited from general practice lists throughout Cambridgeshire. Participants attended one of three study centres (based in Ely, Wisbech and Cambridge), where anthropometric and body composition measures, amongst others, were collected by trained researchers [28]. For this study, home and work addresses for 2,696 working age adults were drawn from the full Fenland Study sample. Exclusions from the full sample were based on incomplete home/work address data (n = 3,475), or living/working very far outside Cambridgeshire (n = 208). Home and work addresses were geocoded based on recorded postcodes and mapped using ArcGIS 10 (ESRI Inc., Redlands, CA). UK postcodes allow for relatively precise geocoding, with each postcode area containing on average only 15 addresses [29]. Fenland study volunteers gave written informed consent and the study was approved by the Cambridge local research ethics committee.

### Food outlet data

After determining the spatial extent of the study participants' home and work postcodes, food outlet locations were obtained from the necessary local councils (n=10) under Freedom of Information requests in November and December 2011 (for more details, see http://www.legislation.gov.uk/ukpga/2000/36/data.pdf). This source of food outlet data is believed to be the most accurate in the UK [30,31]. Food outlets were classified as one of seven food outlet types, derived from Lake *et al*. [30]. The internet, Google Street View, phone calls, some ground truthing and local knowledge were used to define food retailers as either: ‘cafés/coffee shops’, ‘convenience stores’, ‘entertainment, health and leisure’, ‘restaurants’, ‘specialist stores’, ‘supermarkets’ or ‘takeaways’ (includes fast food). In this study we focus on arguably the most salient sources of food within the environment, making up 68% of the foodscape overall: restaurants, takeaways, supermarkets and convenience stores (although the metrics of ‘All Food’ do include food outlets of all types). In the UK, consumption of food in venues such as takeaways and restaurants has increased 29% over the last ten years [32], with these food outlets now constituting over 42% of the eating out market [33]. Meanwhile, 77% of retail food shopping for preparation at home is conducted in large chain supermarkets, whilst the use of convenience stores remains noteworthy (16% of retail food purchasing) [34]. These four types of food outlet have also been the foci of much previous research in the field, and therefore need to be better understood in terms of differential exposure in home and non-home environments.

### Home and work food environment exposure measures

Home and work food outlet density by type was defined as counts of outlets within two definitions of ‘neighbourhood’, both of which were believed to relate to food purchasing behaviours in a previous sample of UK adults [35]. Neighbourhoods were defined: 1) using a 1km street network buffer, matching some working age residents perceptions of ‘neighbourhood’ in the UK [35]; 2) using a 1 mile Euclidean buffer, a distance shown to capture 96% of usual walking destinations from the home in previous work [35]. These definitions of neighbourhood replicate those used in previous studies examining neighbourhood food environment exposure [18,36-38]. Food outlet proximity was defined as the street network distance to the nearest outlet of each type, from home and work addresses [6,7,39]. Street network data were provided by Ordnance Survey as part of their Integrated Transport Network (ITN). Both density and proximity measures have been described elsewhere [7,36,39,40]. Previous research has also suggested that both density and proximity measures are necessary, with weak to moderate correlations observed between these metrics [36], and differential associations with socio-economic status [36] and diet [41].

### Travel route exposure estimation

**Figure 1:**
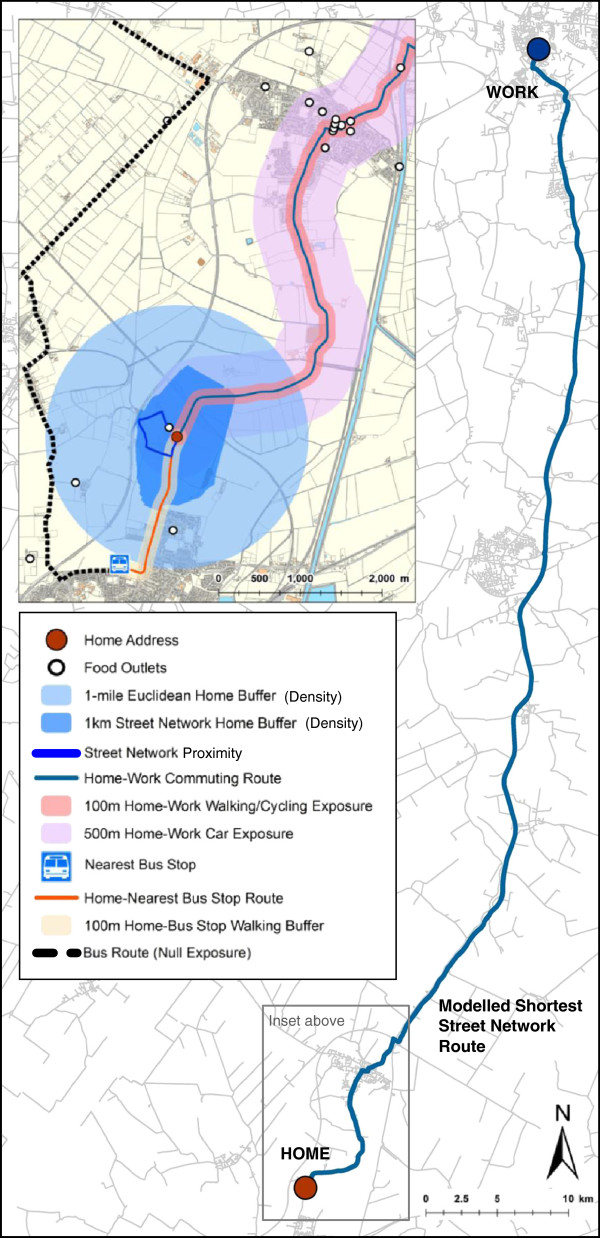
**Exposure metrics created around home address and modelled route to work.** © Crown Copyright/database right 2012. An Ordnance Survey/EDINA supplied service.

Fenland Study participants recorded their travel preferences for commuting to work, both a) by mode – walk, cycle, car, public transport; and b) by frequency – ‘always’, ‘usually’, ‘occasionally’, ‘rarely/never’. Routes for commuting journeys on foot and by bike/car were modelled as the shortest street network distance between homes and workplaces. Exposure along this route was allowed to vary by mode: 100m euclidean buffer around this route for walking/cycling [19,26,27], 500m euclidean buffer around this route for driving. These distances were designed to reflect potential food environment exposure proximal to the route taken. Routes for commuting journeys by public transport were modelled as the shortest street network distance from home to the nearest bus stop, and from the bus stop nearest to work, to work, using public transport node data from the National Public Transport Data Repository (NPTDR) [42]. We assumed these two walking elements of the journey to be made on foot, with route exposure buffered accordingly at 100m. Exposure whilst on the bus was assumed to be null [11]. Figure 1 illustrates the variety of *feature*-*by*-*feature* route and exposure metrics developed for this study [9]. Numbers of food outlets by type were counted within buffered routes; these counts were then expressed per 100m, complementing raw counts. For those individuals who reported multi-modal journeys to work, or reported a single mode but not ‘always’, route exposures were weighted (‘always’=1, ‘usually’= 0.75, ‘occasionally’= 0.25, ‘rarely/never’= 0) [43] and averaged across reported travel modes.

### Statistical analyses

Analysis of variance (ANOVA) was used to test for significant differences in mean density and proximity exposure estimates between homes and workplaces. We also present medians and IQRs for these metrics, at home and work, in Additional file 1: Table S1. Spearman’s Rank correlation co-efficients describe the relative relationships between food environment exposures at home, at work, and whilst commuting. Analyses were conducted using PASW Statistics 18 (PASW Statistics Inc., Chicago, 2009).

## Results

Beyond travel preferences, this paper does not focus on the characteristics of the Fenland Study participants. However, our study sample was representative of the full sample in terms of age (full: mean 46.7 yrs (SD 7.3); study: 46.3 yrs (SD 7.2)), sex (full: 46.1% men, 53.9% women; study: 48.8% men, 51.2% women) and body mass index distributions (full: 26.9 (SD 4.9); study: 26.7 (SD 4.6)), and modal household income, which was more than £40,000 in both the study and overall samples.

**Figure 2:**
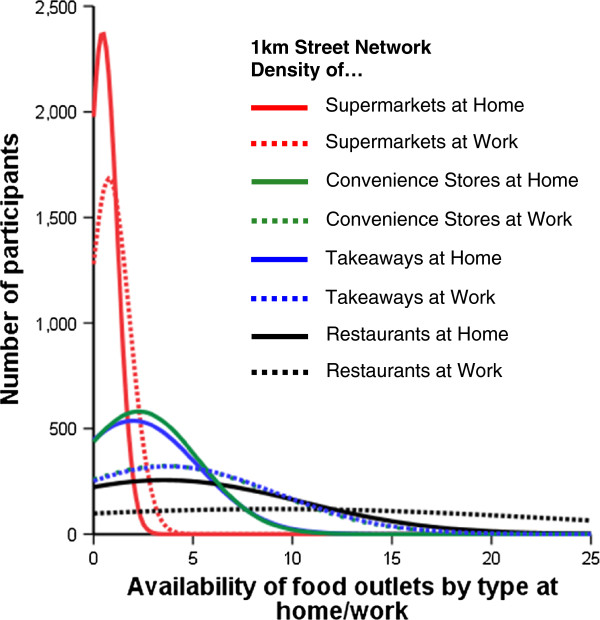
**Home and work food outlet distributions by food outlet type.** Note that work distributions for convenience stores and takeaways overlap.

Densities of all food outlet types, using a 1 mile Euclidean or 1km street network buffer, were greater in work than home neighbourhoods (p < 0.05). Using street network buffers, the density of *all food outlets* was 125% higher at work (Table 1). However, due to the larger size of Euclidean neighbourhoods, actual estimates of exposure using this metric were systematically greater than when using street network neighbourhoods. Among the four outlet types evaluated, percentage differences were greatest for restaurants (155%) and takeaways (80%) in 1km street network buffers. Figure 2 shows density curves for the number of outlets by type at home and work, based on the 1km street network buffer approach. Work distributions are broader and shifted to the right compared to those for the home, for all four outlet types, showing that more people had greater densities of these outlets within their work than their home environments. These differential home-work distributions are supported by medians and IQRs presented in Additional file 1: Table S1.

**Table 1:** **Descriptive statistics for home, work and commuting route**^**a**^**exposures**

		**1km street network density**		**% difference at work**	**1 mile Euclidean density**		**% difference at work**	**Street network proximity (m)**		**% difference at work**	**Commuting route outlet count**	
		**Home**	**Work**		**Home**	**Work**		**Home**	**Work**		**n**		**per 100 metres**
All food outlets	Mean (sd)	12.7 (21.9)	28.6 (41.2)	+125*	68.5 (93.7)	101.7 (126.5)	+48*	631.8 (764.8)	504.7 (738.5)	−20*	61.6 (71.8)	0.9 (1.4)
	Range	161	196		442	444		6849.0	5828.0		627	32.6
Convenience stores	Mean (sd)	2.3 (3.0)	3.8 (5.4)	+66*	10.6 (11.7)	14.3 (15.2)	+35*	1001.3 (1186.5)	953.7 (1119.0)	−5	10.3 (11.7)	0.1 (0.3)
	Range	23	52		48	129		8010.3	9506.3		136	9.00 0.3 (6.8)
Restaurants	Mean (sd)	3.6 (6.8)	9.1 (14.6)	+155*	19.3 (31.0)	31.4 (45.1)	+63*	926.4 (941.1)	849.9 (963.2)	−8*	17.9 (22.8)	0.3 (0.4)
	Range	67	70		155	159		6892.1	6773.6		195	7.87
Supermarkets	Mean (sd)	0.5 (0.7)	0.8 (1.0)	+71*	2.2 (2.9)	3.1 (3.4)	+40*	3040.5 (3122.3)	2237.6 (2653.0)	−26*	1.9 (2.2)	0.02 (0.05)
	Range	4	5		14	13		14371.8	15442.4		15	1.69
Takeaways	Mean (sd)	2.0 (3.3)	3.6 (5.4)	+80*	9.5 (10.8)	13.0 (13.0)	+36*	1353.3 (1508.8)	1265.3 (1478.7)	−7*	8.8 (10.2)	0.1 (0.2)
	Range	21	34		47	62		10054.3	10486.3		95	6.18
Sample size (n)		2696	2696		2696	2696		2696	2696		2351	2351

* Significant difference (ANOVA, p<0.05) between home and work locations.

^a^ Commuting routes were defined according to the shortest street network distance between home and workplace, accounting for travel mode.

Table 1 also shows that proximity to the majority of food outlet types was also significantly greater (shorter distances) around workplaces than homes (p < 0.05). Supermarkets showed the largest difference, 26% closer (approximately 1km) to the workplace than to home. Other differences in proximity were less marked (outlets around work 6-8% closer than those outlets around home). Proximity to convenience stores was not significantly different at work relative to home.

**Figure 3:**
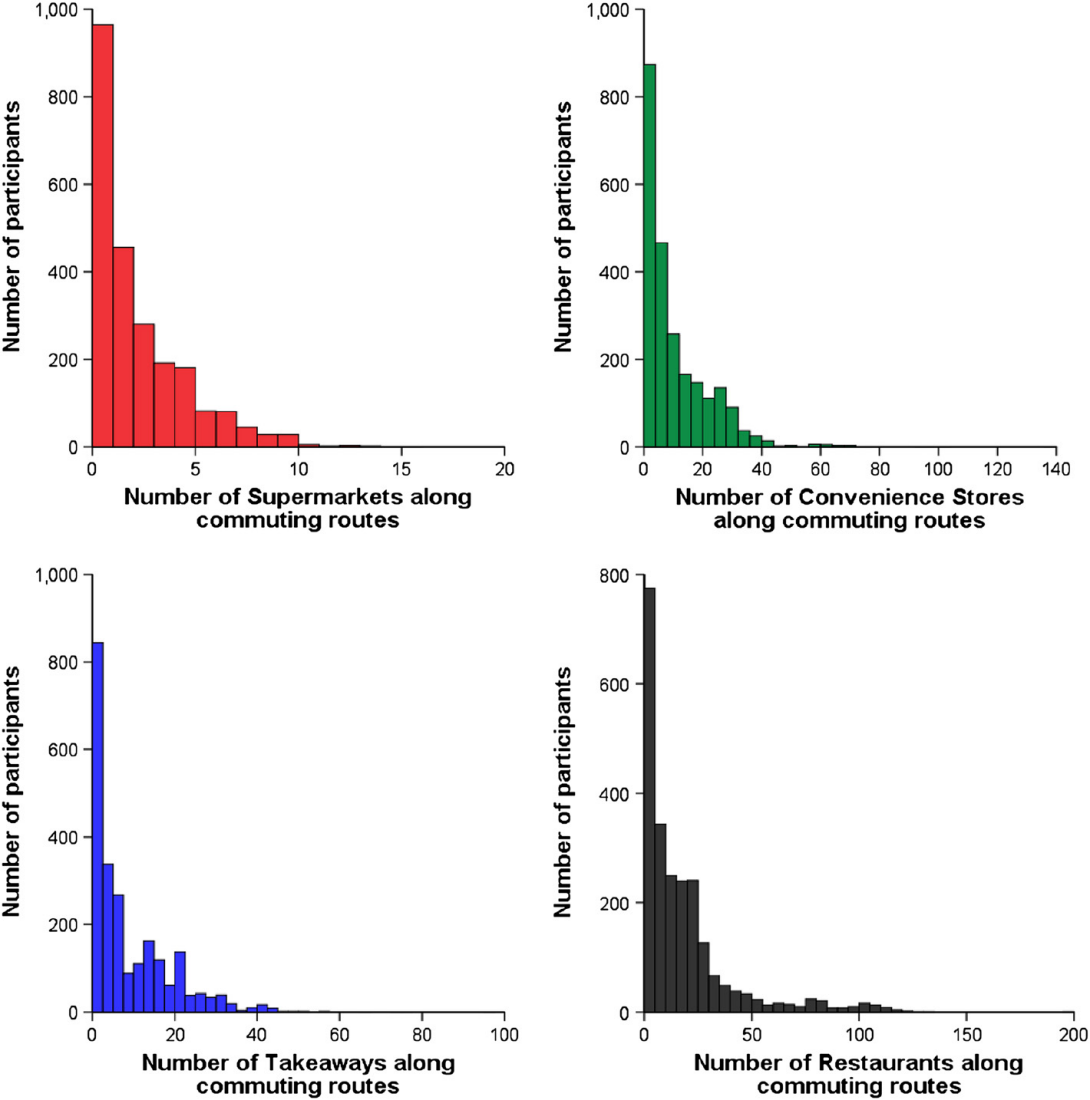
Histograms showing commuting route exposure by food outlet type.

Average commuting route exposures varied by food outlet type (Table 1). Participants were exposed to a maximum of 627 food outlets of all types along their journey to work, an effective availability of up to 32.6 outlets for every 100 metres travelled. Exposure to all types of food outlet was also substantial along these routes, as illustrated by the distributions in Figure 3. Route exposure to over 20 takeaway food outlets, for example, was not uncommon, with one individual potentially exposed to 95 takeaway food outlets. Participants were exposed to seven times more convenience stores per 100 m than supermarkets when making this journey, on average. Finally, matching the particularly elevated availability of restaurants around workplaces relative to the home, participants would be most highly exposed to this type of food outlet whilst commuting, too (mean, 17.9 outlets).

**Figure 4:**
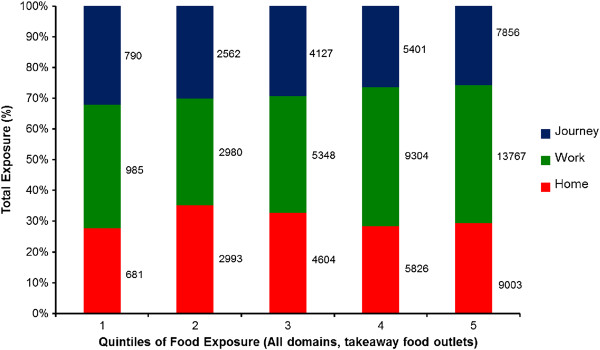
**Percentage contribution by domain (home, work, journey), to daily takeaway food outlet environmental exposure, stratified by quintiles of daily takeaway outlet exposure**^**a**^**.**

Building upon these descriptive statistics, it is also important to understand the percentage contribution made by each exposure domain to overall food outlet exposure. The contribution to food outlet exposure from home, work and commuting route environments varied greatly on a person-to-person basis. Figure 4 shows average percentage contribution by domain (home, work, journey), to combined home/work/journey takeaway food outlet environmental exposure, stratified by quintiles of combined exposure. The percentage contributions to exposure from the home remain relatively stable across quintiles, varying from 35% to 27% of total exposure. These percentage contributions are similar to those from commuting routes, which themselves tend to decline in contribution as workplace exposure increases in quintile five. Overall, percentage contributions from workplace food environments to takeaway foodscape exposure increase as total foodscape exposure increases from 40% to 45% (contributing as little as 35% in quintile 2). These findings are largely consistent by outlet type (similar figures for supermarkets, convenience stores and restaurants, shown in Additional file 2: Figure S1).

To examine the degree of agreement between relative foodscape exposure at home, at work, and whilst commuting, we used rank correlations. Spearman’s rank correlations of foodscape exposure (by outlet type) across home, commuting and work domains are presented in Table 2, using 1 mile Euclidean home and work definitions of neighbourhood. Throughout, correlations of the home environment with commuting, of the home environment with work, and of the commuting environment with work, are weak or null, across all types of food outlet exposure. These findings were highly consistent across other measures of street network food outlet density, and proximity (results not presented), and suggest that there was little relationship at the individual level between relative food environment exposure at home and relative food environment exposures in work and commuting domains.

**Table 2:** Spearman’s rank correlations of food outlet counts (by type) across exposure domains

**Food outlet type**	**Home density x commuting density**^**a**^	**Home density x work density**^**a**^	**Commuting density x work density**^**a**^
All Outlets	−0.010	0.340*	0.201*
Convenience Stores	−0.088*	0.341*	0.150*
Restaurants	0.023	0.355*	0.232*
Supermarkets	−0.001	0.355*	0.206*
Takeaways	−0.002	0.291*	0.209*

*P<0.001.

^a^1 mile Euclidean density presented; results consistent across street network density and proximity metrics.

## Discussion

Our study was motivated by the question of how food environments differed between homes, workplaces and along commuting routes between home and work. We used data from a sample of working residents in the East of England to apply methods for characterising foodscapes in these three exposure domains, which importantly accounted for both travel mode and frequency. Our paper goes beyond most of the published food environment literature through demonstrating very different foodscapes around homes and workplaces, as well as a potentially substantial exposure contribution from commuting routes. On average, work and commuting domains contributed to total foodscape exposure at least equally to the residential contribution. Moreover, levels of relative exposure between home, work and commuting route environments were poorly correlated.

On average, density of and proximity to all food outlet types was significantly greater at work than at home, regardless of the neighbourhood definition on which density was based. Similar results have been found elsewhere [11,18]. The greatest difference in outlet density between home and work was found for restaurants and takeaways. Workplace environments also provided participants with significantly greater proximity (shorter distances) to these outlets. Considering the general unhealthiness of foods consumed away from the home [23], increased exposure to these particular types of food outlets at work might be considered a public health concern. These differences between homes and work place exposures were even more pronounced in a sub-sample of Fenland participants who lived in rural but worked in urban areas throughout the study region (data not shown). Whilst adult commuting route exposures have not been directly addressed in the literature, previous small scale studies investigating wider activity space exposures have largely found them at least equal to those experienced in the neighbourhood of residence, consistent with our results [17,24].

Supermarket density and supermarket proximity were also significantly increased at work, suggesting greater exposure to a wide range of nutritious foods at a variety of price points in this particular setting [21,22,44]. In line with previous findings [17], 77% of this sample had greater supermarket density within 1 mile of their workplace, than within 1 mile of their home. However, higher supermarket exposure around work places might not be associated with utilisation of these outlets if individuals did not have a car at work (e.g., those who commuted by bus). Other studies have found that car use plays a pivotal role in perceived supermarket access [34]. *Particular types* of food outlet may be especially important then, in *particular settings*. Increased density and proximity of takeaway food outlets at work may prove particularly influential on behaviour. For example, during a lunch period at work, an individual might need to purchase food, but time constraints might restrict how far he/she may travel to acquire it [45], thereby strengthening the link between the proximal foodscape and utilisation. Similarly, when commuting home from work, individuals may place a high priority on convenient, ready prepared meals, therefore strengthening the exposure to restaurants and takeaways in this setting. These spatio-temporal imperatives may be less pressing, and hence these exposures less pertinent in the residential setting, particularly for individuals who have use of a car. Understanding that behaviours are likely to vary between settings, and that meanwhile, food environment exposures also differ dramatically along the same lines, will be critical in determining accurate individual-environment associations in future work.

### Implications for research

Overall it is clear that focussing solely on home locations would have resulted in a severe mis-estimation of foodscape exposure. We presented evidence that percentage contributions to food environment exposure (of all types) were similar if not greater in non-home settings, compared to around the home. Furthermore, evident in terms of takeaway food outlet density, was the trend for those with the greatest exposure to takeaway food outlets to be mostly exposed at work. This is an important point as it means that relying solely on residential takeaway food outlet exposure would particularly underestimate total exposure for those most exposed to total takeaway outlet numbers. The ability to detect accurate associations between environmental exposures and health outcomes would therefore be compromised.

Furthermore, this exploratory work provides little evidence that those with relatively low home exposure also experienced relatively low commuting and work exposures. In actuality, the low correlation between the domains would suggest that the levels of exposure across the three domains varied randomly on a person-to-person basis. This finding corroborates results elsewhere in relation to alcohol retailers [46] and fast food outlets [24], with exposure assessed to the latter using GPS, but in a small sample. These different levels of relative exposure across environmental settings would be particularly problematic if only residential exposure were to be assessed [17]. The findings suggest that relying solely on residential foodscape exposures would likely lead to misclassification of total foodscape exposure and therefore attenuated associations between exposures and diet or health outcomes. In light of this, the reporting of null associations between foodscapes and diet or health outcomes in the literature might be attributed to underestimation of total exposure and a neglect of particularly salient domains of exposure [47,48].

Whilst the secondary datasets upon which the field often relies usually do not contain data on both home and work locations of participants, accounting additionally for non-residential exposures in order to address the ‘neighbourhoods, mobility, and health triad’ [9], and to avoid the ‘residential trap’ [10], is surely one direction in which the field of obesogenic environment studies needs to progress. Such weaknesses can begin to be resolved early in the research process, through collecting data that will help us better address our research questions.

### Implications for policy

Whilst we acknowledge that access to food extends beyond simply spatial concerns, including for example economic considerations [44], current and emerging government strategies to promote healthier eating in the USA and the UK are heavily focussed on spatial access to food outlets. At best however, such strategies are apparently predicated on a limited evidence base that has conceptualised food environments primarily around residential neighbourhoods. To justify the environmental changes emerging from these policy initiatives, such as restricting the locations of hot ‘fast food’ type retailers [49,50], greater consensus for an individual-environment association, based firmly on a more complete comprehension of environmental exposure, needs to be achieved. Although we do not examine associations between more complete estimations of exposure and dietary outcomes/body weight in this paper, based on the extent of these exposures, such research is required. Furthermore, by demonstrating domain specific associations with pertinent dietary outcomes, it may be possible to better direct policies with the aim of improving health. Work environments, for example, might be particularly related to the consumption of particular foods, but the evidence base currently focussed on residential exposure is not best placed to assess this association. Ultimately, we aim for this study to contribute to a more comprehensive evidence base that can inform public policy.

### Methodological considerations and limitations

Limitations of this study include the uncertainty of modelling commuting routes to work based on the shortest street network distance. Although there is precedent for this approach in the literature [19,26,27], there are myriad reasons why participants may not have followed these imputed routes, potentially resulting in actual foodscape exposures different from those estimated here. For example, to ‘trip chain’ a gym or some other visit into the journey home from work, which is increasingly likely for those commuting longer distances [34]. However, individuals may at least use these assumed routes and experience their associated exposures on one leg of their round trip to/from work, or for a segment of one of these journeys. Routes produced by our GIS software also align closely with those suggested by commercial mapping tools, such as Google Maps, providing another indication of their credibility. Furthermore, using assumed routes may limit the effects of confounding, potentially emerging from individuals’ selecting to travel certain routes precisely because of preferential food access [9]. GPS and/or travel diaries have the potential to better capture the specifics (the precise route used to/from work, for example, which may not follow the shortest route) and extent of ‘activity spaces’ [11], including for example foodscape exposure during leisure time, which we are unable to account for. Although there is some precedent in the literature for capturing spatial polygamy [9] through the use of these approaches [11,17,24,46,51], further work is required in this regard to fully understand residential/non-residential, and domain specific food environment exposures.

Whilst dietary behaviours are theoretically intertwined with foodscapes, exposure does not necessarily equate to utilisation, with individuals not compelled to shop at their most spatially convenient food outlet. Whilst significant differences in food environments between home, work and commuting routes have been demonstrated in this paper, which should in turn translate into more realistic exposure estimates, associations between individuals’ behaviours and their activity space environments are yet to be examined. We also acknowledge that considerations other than spatial (for example, economic) are likely to be important behavioural determinants. Whilst we note that the Fenland Study is not strictly a representative sample of the study area population, its characteristics are typical of those we might expect of this region of the UK (predominantly white British and relatively affluent). Moreover, we consider our sub-sample to be representative of the full Fenland Study sample, and provide evidence for this assertion, however we acknowledge that our sample was predominantly derived based on the completeness of home and work address data.

## Conclusions

This study introduced a novel environmental exposure case study, focussed on different sources of food, and based on home, work and commuting route environments that were sensitive to travel preferences. Previous studies have very much focussed on the home food environment only, despite the fact that behaviours related to consumption occur outside this setting. Our findings indicated that home and work foodscapes were very different and unsystematically different, that commuting routes may constitute an important exposure, and that cumulative daily exposure might far outweigh that experienced in the residential neighbourhood alone, especially for the most exposed. We suggest that the importance of food outlets in determining behaviours may be both outlet type and location specific. Future work will consider how important these different foodscapes, and cumulative food availability are in explaining the social patterning of diet and health.

## Competing interests

The authors declare they have no competing interests.

## Authors' contributions

The study design was jointly devised by TB and PM. TB was responsible for data collection from local councils, and led on data analysis, in consultation with PM. TB and PM drafted the manuscript together. Both authors read and approved the final manuscript.

## Supplementary Material

Additional file 1: Table S1 Medians and interquartile ranges (IQRs) for home, work and commuting route^a^ exposure. Click here for file

Additional file 2: Figure S1 Percentage contribution by domain (home, work, journey), to daily food outlet environmental exposure by type (see horizontal axis title), stratified by quintiles of daily food outlet exposure by type^a^. Click here for file

## References

[B1] Lake AA Townshend T Obesogenic environments: exploring the built and food environment Perspect Public Health 2006 126 262 267 10.1177/1466424006070487 17152319

[B2] Townshend T Lake AA Obesogenic urban form: theory, policy and practice Health Place 2009 15 909 916 19201641 10.1016/j.healthplace.2008.12.002

[B3] Glanz K Sallis JF Saelens BE Frank LD Healthy nutrition environments: concepts and measures Am J Health Promot 2005 19 330 333 15895534 10.4278/0890-1171-19.5.330

[B4] Egger G Swinburn B An 'ecological approach' to the obesity pandemic Br Med J 1997 315 477 480 9284671 10.1136/bmj.315.7106.477 PMC2127317

[B5] Boone-Heinonen J Gordon-Larsen P Obesogenic environments in youth: concepts and methods from a longitudinal national sample Am J Prev Med 2012 42 e37 e46 22516502 10.1016/j.amepre.2012.02.005 PMC3382037

[B6] Caspi CE Sorensen G Subramanian SV Kawachi I The local food environment and diet: a systematic review Health Place 2012 18 1172 1187 22717379 10.1016/j.healthplace.2012.05.006 PMC3684395

[B7] Charreire H Casey R Salze P Simon C Chaix B Banos A Badariotti D Weber C Oppert JM Measuring the food environment using geographical information systems: a methodological review Public Health Nutr 2010 13 1773 1785 20409354 10.1017/S1368980010000753

[B8] Lake AA Townshend T Alvanides S Obesogenic environments: complexities, perceptions and objective measures 2010 Oxford: Blackwell Publishing Ltd

[B9] Chaix B Kestens Y Perchoux C Karusisi N Merlo J Labadi K An interactive mapping tool to assess individual mobility patterns in neighbourhood studies Am J Prev Med 2012 43 440 450 22992364 10.1016/j.amepre.2012.06.026

[B10] Chaix B Geographic life environments and coronary heart disease: a literature review, theoretical contributions, methodoligical updates, and a research agenda Annu Rev Public Health 2009 30 81 105 19705556 10.1146/annurev.publhealth.031308.100158

[B11] Kestens Y Lebel A Daniel M Thériault M Pampalon R Using experienced activity spaces to measure foodscape exposure Health Place 2010 16 1094 1103 20667762 10.1016/j.healthplace.2010.06.016

[B12] Cummins S Commentary: Investigating neighbourhood effects on health - avoiding the 'Local Trap' Int J Epidemiol 2007 36 355 357 17376797 10.1093/ije/dym033

[B13] Kwan MP From place-based to people-based exposure measures Soc Sci Med 2009 69 1311 1313 19665828 10.1016/j.socscimed.2009.07.013

[B14] Lovasi GS Vernez Moudon A Pearson AL Hurvitz PM Larson EB Siscovick DS Berke EM Lumley T Psaty BM Using built environment characteristics to predict walking for exercise Int J Health Geogr 2008 7 1 13 18312660 10.1186/1476-072X-7-10 PMC2279119

[B15] Jones A Bentham P Foster C Hillsdon M Panter J Foresight Tackling Obesities: Future choices Obesogenic environments - Evidence review 2007 Office of Science and Innovation

[B16] White E Armstrong BK Saracci R Principles of measurement in epidemiology: collecting, evaluating, and improving measures of disease risk factors 2008 2 Oxford: Oxford University Press

[B17] Hurvitz PM Vernez Moudon A Home versus nonhome neighbourhood: quantifying differences in exposure to the built environment Am J Prev Med 2012 42 411 417 22424255 10.1016/j.amepre.2011.11.015 PMC3318915

[B18] Jeffery RW Baxter J McGuire M Linde J Are fast food restaurants an environmental risk factor for obesity? Int J Behav Nutr Phys Act 2006 3 1 6 16436207 10.1186/1479-5868-3-2 PMC1397859

[B19] Harrison F Jones AP van Sluijs EMF Cassidy A Bentham G Griffin SJ Environmental correlates of adiposity in 9–10 year old children: considering home and school neighbourhoods and routes to school Soc Sci Med 2011 72 1411 1419 21481505 10.1016/j.socscimed.2011.02.023 PMC3759221

[B20] An R Sturm R School and residential neighbourhood food environment and diet among California youth Am J Prev Med 2012 42 129 135 22261208 10.1016/j.amepre.2011.10.012 PMC3298889

[B21] Zenk SN Schultz AJ Israel BA James SA Bao S Wilson ML Neighbourhood racial composition, neighbourhood poverty, and the spatial accessibility of supermarkets in metropolitan Detroit Am J Public Health 2005 95 660 667 15798127 10.2105/AJPH.2004.042150 PMC1449238

[B22] Burns CM Inglis AD Measuring food access in Melbourne: Access to health and fast foods by car, bus and foot in an urban municipality in Melbourne Health Place 2007 13 877 885 17470408 10.1016/j.healthplace.2007.02.005

[B23] Lachat C Nago E Verstraeten R Roberfroid D Van Camp J Kolsteren P Eating out of home and its association with dietary intake: a systematic review of the evidence Obes Rev 2012 13 329 346 22106948 10.1111/j.1467-789X.2011.00953.x

[B24] Zenk SN Schulz AJ Matthews SA Odoms-Young A Wilbur J Wegrzyn L Gibbs K Braunschweig C Stokes C Activity space environment and dietary and physical activity behaviours: a pilot study Health Place 2011 17 1150 1161 21696995 10.1016/j.healthplace.2011.05.001 PMC3224849

[B25] Duncan MJ Mummery WK GIS or GPS? A comparison of two methods for assessing route taken during active transport Am J Prev Med 2007 33 51 53 17572312 10.1016/j.amepre.2007.02.042

[B26] Panter JR Jones AP van Sluijs EMF Griffin SJ Neighbourhood, route, and school environments and children's active commuting Am J Prev Med 2010 38 268 278 20171528 10.1016/j.amepre.2009.10.040 PMC3819023

[B27] Timperio A Ball K Salmon J Roberts R Giles-Corti B Simmons D Baur LA Crawford D Personal, family, social, and environmental correlates of active commuting to school Am J Prev Med 2006 30 45 51 16414423 10.1016/j.amepre.2005.08.047

[B28] De Lucia Rolfe E Loos RJF Druet C Stolk RP Ekelund U Griffin SJ Forouhi NG Wareham NJ Ong KK Association between birth weight and visceral fat in adults Am J Clin Nutr 2010 92 347 352 20519560 10.3945/ajcn.2010.29247

[B29] Smith D Cummins S Clark C Stansfeld S Does the local food environment around schools affect diet? Longitudinal associations in adolescents attending secondary schools in East London BMC Publ Health 2013 13 1 10 10.1186/1471-2458-13-70 PMC3567930 23347757

[B30] Lake AA Burgoine T Greenhalgh F Stamp E Tyrrell R The foodscape: classification and field validation of secondary data sources Health Place 2010 16 666 673 20207577 10.1016/j.healthplace.2010.02.004

[B31] Lake AA Burgoine T Stamp E Grieve R The foodscape: classification and field validation of secondary data sources across urban/rural and socio-economic classifications Int J Behav Nutr Phys Act 2012 9 3 12 22472206 10.1186/1479-5868-9-37 PMC3341208

[B32] Food: an analysis of the issueshttp://webarchive.nationalarchives.gov.uk/+/http://www.cabinetoffice.gov.uk/media/cabinetoffice/strategy/assets/food/food_analysis.pdf

[B33] Food matters: towards a strategy for the 21st centuryhttp://webarchive.nationalarchives.gov.uk/+/http:/www.cabinetoffice.gov.uk/media/cabinetoffice/strategy/assets/food/food_matters1.pdf

[B34] White M Bunting J Williams L Raybould S Adamson A Mathers J Do 'food deserts' exist? A multi-level, geographical analysis of the relationship between retail food access, socio-economic position and dietary intake. Final Report to the Food Standards Agency 2004

[B35] Smith G Gidlow C Davey R Foster C What is my walking neighbourhood? A pilot study of English adults' definitions of their local walking neighbourhoods Int J Behav Nutr Phys Act 2010 7 1479 5868 10.1186/1479-5868-7-34 PMC2873577 20459636

[B36] Apparicio P Cloutier MS Shearmur R The case of Montreal's missing food deserts: evaluation of accessibility to food supermarkets Int J Health Geogr 2007 6 1 13 17295912 10.1186/1476-072X-6-4 PMC1803774

[B37] Smoyer-Tomic KE Spence JC Amrhein C Food deserts in the prairies? Supermarket accessibility and neighbourhood need in Edmonton, Canada Prof Geogr 2006 58 307 326

[B38] Larsen K Gilliland J Mapping the evolution of 'food deserts' in a Canadian city: Supermarket accessibility in London, Ontario, 1961–2005 Int J Health Geogr 2008 7 16 32 18423005 10.1186/1476-072X-7-16 PMC2387138

[B39] Thornton LE Pearce JR Kavanagh AM Using geographic information systems (GIS) to assess the role of the built environment in influencing obesity: a glossary Int J Behav Nutr Phys Act 2011 8 10 21722367 10.1186/1479-5868-8-71 PMC3141619

[B40] Sparks AL Bania N Leete L Comparative approaches to measuring food access in urban areas: the case of Portland, Oregon Urban Studies 2011 48 1715 1737 21954485 10.1177/0042098010375994

[B41] Thornton LE Pearce JR Macdonald L Lamb KE Ellaway A Does the choice of neighbourhood supermarket access measure influence associations with individual-level fruit and vegetable consumption? A case study from Glasgow Int J Health Geogr 2012 11 10 22839742 10.1186/1476-072X-11-29 PMC3460757

[B42] National Public Transport Data Repository (NPTDR) http://data.gov.uk/dataset/nptdr

[B43] Besson H Brage S Jakes RW Ekelund U Wareham NJ Estimating physical activity energy expenditure, sedentary time, and physical activity intensity by self-report in adults Am J Clin Nutr 2010 91 106 114 19889820 10.3945/ajcn.2009.28432

[B44] Drewnowski A Aggarwal A Hurvitz PM Monsivais P Vernez Moudon A Obesity and supermarket access: proximity or price? Am J Public Health 2012 102 74 80 10.2105/AJPH.2012.300660 PMC3464835 22698052

[B45] Matthews AA Vernez Moudon A Daniel M Work group II: using geographic information systems for enhancing research relevant to policy on diet, physical activity, and weight Am J Prev Med 2009 36 S171 S176 19285210 10.1016/j.amepre.2009.01.011

[B46] Basta LA Richmond TS Wiebe DJ Neighbourhoods, daily activities, and measuring health risks experienced in urban environments Soc Sci Med 2010 71 1943 1950 20980088 10.1016/j.socscimed.2010.09.008 PMC2982925

[B47] Feng J Glass TA Curriero FC Stewart WF Schwartz BS The built environment and obesity: a systematic review of the epidemiologic evidence Health Place 2010 16 175 190 19880341 10.1016/j.healthplace.2009.09.008

[B48] Rainham D McDowell I Krewski D Sawada M Conceptualizing the healthscape: contributions of time geography, location technologies and spatial ecology to place and health research Soc Sci Med 2010 70 668 676 19963310 10.1016/j.socscimed.2009.10.035

[B49] Greater London Authority Takeaways toolkit: tools, interventions and case studies to help local authorities develop a response to the health impacts of fast food takeaways 2012 London: Chartered Institute of Environmental Health

[B50] Waltham Forest SPD Waltham Forest SPD - hot food takeaway shops 2009 Borough of Waltham Forest: London

[B51] Coombes E van Sluijs E Jones A **Is environmental setting associated with the intensity and duration of children's physical activity? Findings from the SPEEDY GPS study.** Health Place 2013 20 62 65 23376730 10.1016/j.healthplace.2012.11.008 PMC3591252

