# Supplementary material for: Characterising food environment exposure at home, at work, and along commuting journeys using data on adults in the UK
Source: Int J Behav Nutr Phys Act. 2013 Jun 27;10:85. doi: 10.1186/1479-5868-10-85 (PMC3720205; doi:10.1186/1479-5868-10-85)
Supplement: Additional file 1: Table S1 — Medians and interquartile ranges (IQRs) for home, work and commuting routea exposure. [file 1479-5868-10-85-S1.doc]

|  |  | **1km Street Network Density** | | **% difference at work** | **1mile Euclidean Density** | | **% difference at work** |  | **Street Network Proximity (m)** | | **% difference at work** | **Commuting route outlet count** | |
| --- | --- | --- | --- | --- | --- | --- | --- | --- | --- | --- | --- | --- | --- |
|  |  | **Home** | **Work** |  | **Home** | **Work** |  | **Home** | | **Work** |  | **n** | **per 100 metres** |
| All Food Outlets | Median (IQR) | 5  (1-11) | 7  (1-44) | +40 | 25  (6-95) | 39  (11-110) | +56 | 440.9  (221-750) | | 265.5  (0-707) | -40 | 34.0  (11-90) | 0.004  (0.002-0.010) |
| Convenience Stores | Median (IQR) | 1  (0-3) | 1  (0-5) | 0 | 7  (2-13) | 9  (2-26) | +29 | 654.3  (370-1058) | | 590.3  (278-1224) | -10 | 6.0  (2-15) | 0.001  (0.000-0.001) |
| Restaurants | Median (IQR) | 1  (0-3) | 2  (0-14) | +100 | 5  (2-22) | 9  (3-24) | +80 | 674.1  (383-1098) | | 551.0  (208-1114) | -18 | 10.3  (3-23) | 0.001  (0.000-0.003) |
| Supermarkets | Median (IQR) | 0  (0-1) | 0  (0-1) | 0 | 1  (0-3) | 2  (0-4) | +100 | 1617.3  (830-4908) | | 1091.6  (587-2568) | -33 | 1.0  (0-3) | 0.000  (0.000-0.000) |
| Takeaways | Median (IQR) | 1  (0-2) | 1  (0-5) | 0 | 5  (1-14) | 9  (2-22) | +80 | 863.5  (486-1468) | | 781.0  (336-1595) | -10 | 4.7  (1-14) | 0.001  (0.000-0.001) |
| Sample size (n) | | 2696 | 2696 |  | 2696 | 2696 |  | 2696 | | 2696 |  | 2351 | 2351 |
| ^a^ Commuting routes were defined according to the shortest street network distance between home and workplace, sensitive to travel mode. | | | | | | | | | | | | | |

**Table 2: Medians and interquartile ranges (IQRs)** for home, work and commuting route^a^ exposure.
