# Supplementary material for: Characterising food environment exposure at home, at work, and along commuting journeys using data on adults in the UK
Source: Int J Behav Nutr Phys Act. 2013 Jun 27;10:85. doi: 10.1186/1479-5868-10-85 (PMC3720205; doi:10.1186/1479-5868-10-85)
Supplement: Additional file 2: Figure S1 — Percentage contribution by domain (home, work, journey), to daily food outlet environmental exposure by type (see horizontal axis title), stratified by quintiles of daily food outlet exposure by typea. [file 1479-5868-10-85-S2.doc]

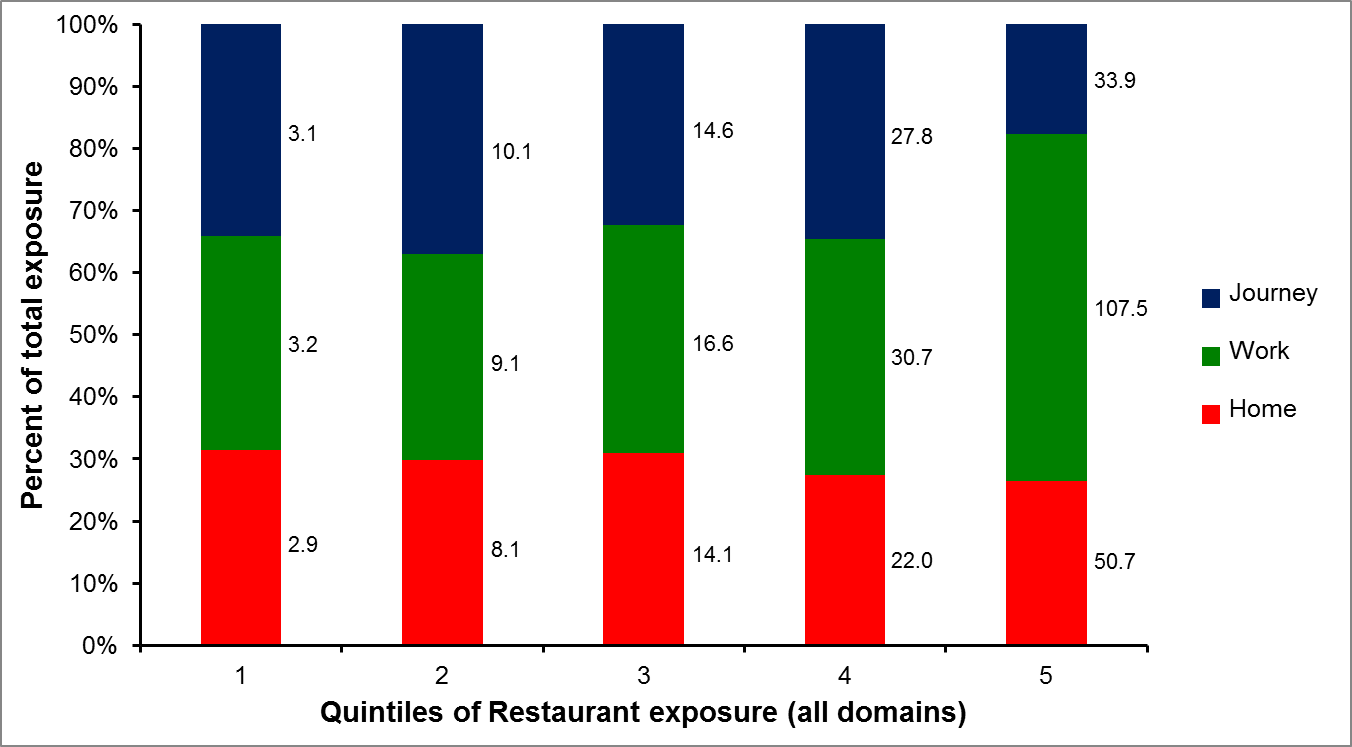


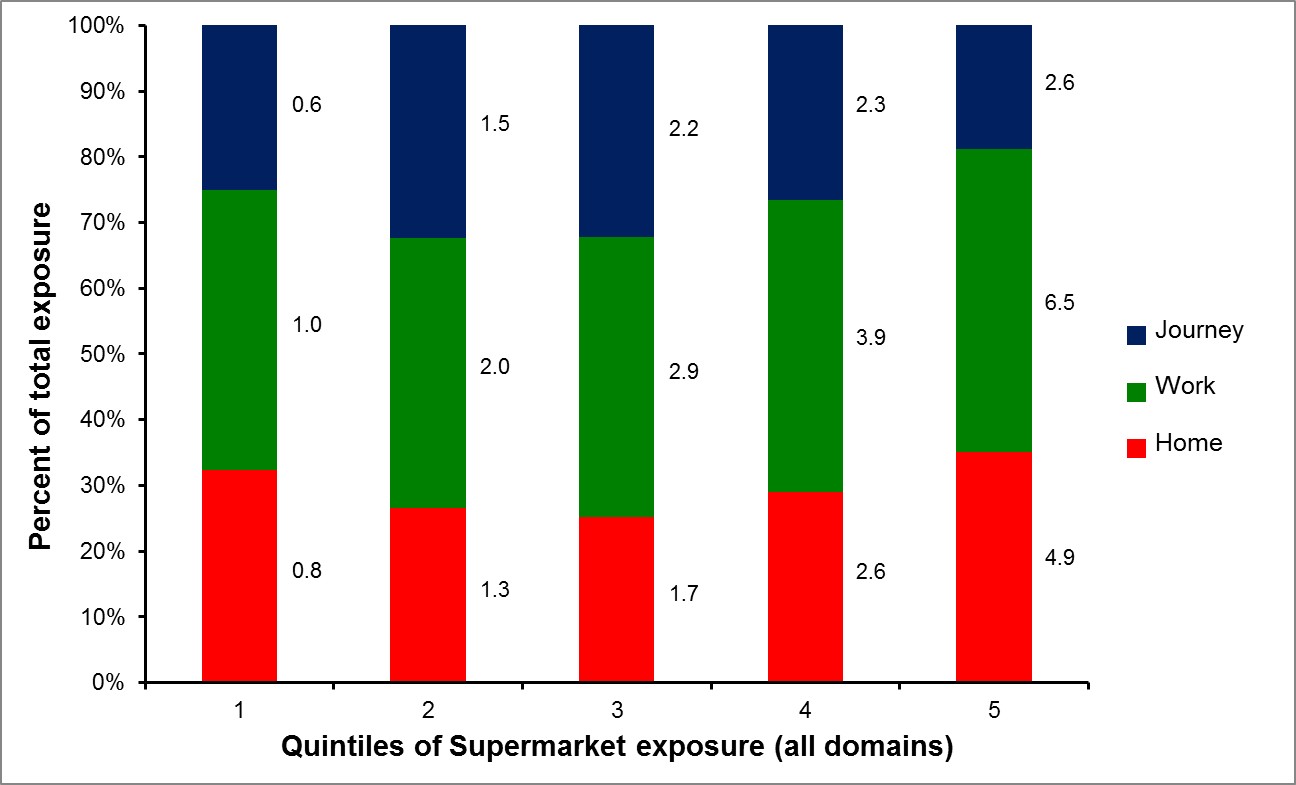


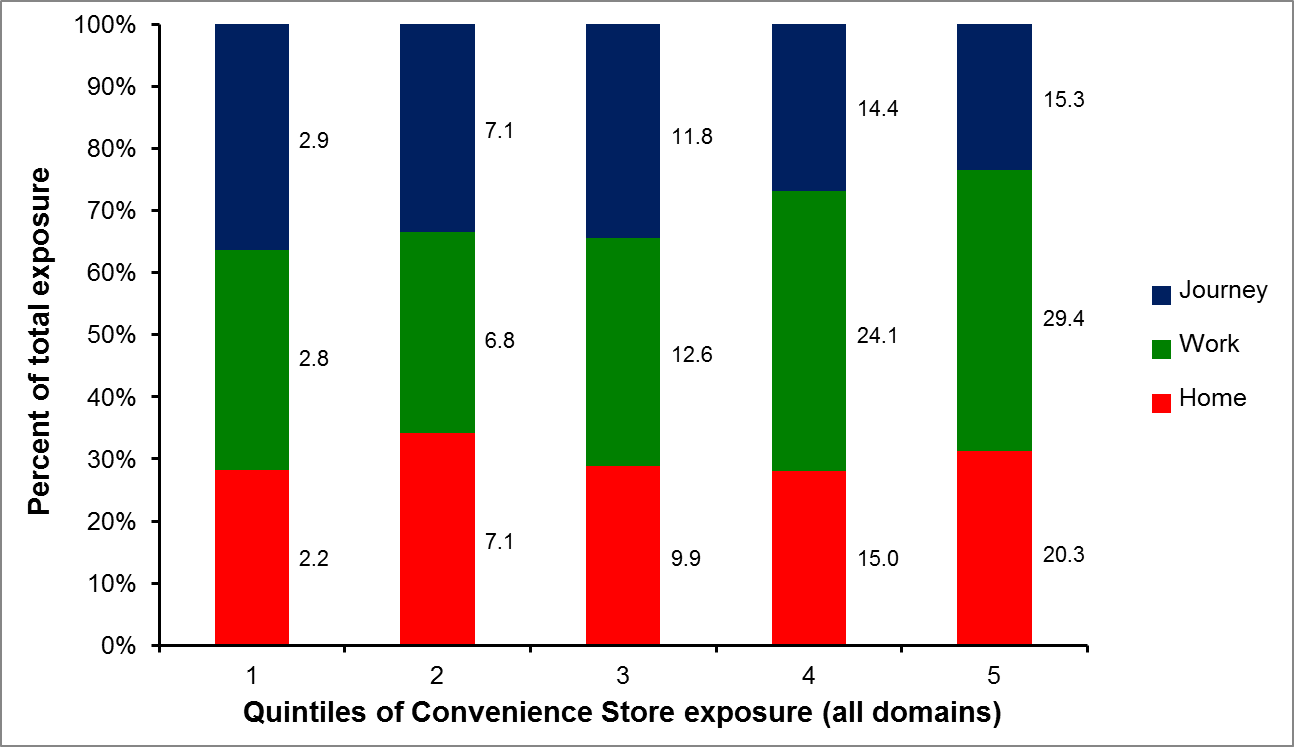


^a^ Mean numbers (per person) of outlets per domain, per quintile of combined home, work and journey outlet exposure, also presented alongside bars.

**Figure 5: Percentage contribution by domain** (home, work, journey), to daily food outlet environmental exposure by type (see horizontal axis title), stratified by quintiles of daily food outlet exposure by type^a^.
